# Controllable Sequence Editing for Counterfactual Generation

**Published:** 2025-02-05

**Authors:** Michelle M. Li, Kevin Li, Yasha Ektefaie, Shvat Messica, Marinka Zitnik

**Affiliations:** 1 Harvard Medical School, Family Living Laboratory Collaboration at Harvard Medical School and Clalit Research Institute; 2 The Ivan and Francesca Berkowitz Family Living Laboratory Collaboration at Harvard Medical School and Clalit Research Institute; 3 Massachusetts Institute of Technology; 4 Kempner Institute for the Study of Natural and Artificial Intelligence at Harvard University.

## Abstract

Sequence models generate counterfactuals by modifying parts of a sequence based on a given condition, enabling reasoning about “what if” scenarios. While these models excel at conditional generation, they lack fine-grained control over when and where edits occur. Existing approaches either focus on univariate sequences or assume that interventions affect the entire sequence globally. However, many applications require precise, localized modifications, where interventions take effect only after a specified time and impact only a subset of co-occurring variables. We introduce Clef, a controllable sequence editing model for counterfactual reasoning about both immediate and delayed effects. Clef learns temporal concepts that encode how and when interventions should influence a sequence. With these concepts, Clef selectively edits relevant time steps while preserving unaffected portions of the sequence. We evaluate Clef on cellular and patient trajectory datasets, where gene regulation affects only certain genes at specific time steps, or medical interventions alter only a subset of lab measurements. Clef improves immediate sequence editing by up to 36.01% in MAE compared to baselines. Unlike prior methods, Clef enables one-step generation of counterfactual sequences at any future time step, outperforming baselines by up to 65.71% in MAE. A case study on patients with type 1 diabetes mellitus shows that Clef identifies clinical interventions that shift patient trajectories toward healthier outcomes.

## 1. Introduction

Counterfactual thinking is a fundamental objective in biology and medicine (Lee & Topol, 2024). “What if” scenarios are critical for reasoning about the underlying mechanisms of a cell, patient, disease, and drug, and each decision can have tangible impact (Bunne et al., 2024; Lee & Topol, 2024): *What if we treat the cells with the candidate drug every hour or every 24 hours? What if we perform the surgery on the patient today or next year?* We should not only reason about the choice of the counterfactual condition (e.g., drug, surgery), but also its timing (e.g., when and how frequent). Thus, counterfactual generation requires precise and context-specific edits that adhere to temporal and structural constraints. For example, prescribing a medication to a patient should result in changes to the patient’s trajectory only after the intervention time (i.e., the medical history prior to intervention should be unaffected to preserve temporal causality) and on only the relevant variables that are specific to the context of the intervention (i.e., the measurements unaffected by the intervention should be preserved).

Generative models in the language and vision domains enable precise editing guided by a description, such as textual prompts or condition tokens (Zhang et al., 2023b; Gao et al., 2023; Ravi et al., 2024; Gong et al., 2024; Niu et al., 2024; Gu et al., 2024; Zhou et al., 2024). These models are designed to gain more comprehensive (i.e., global) and precise (i.e., local) control over the generation of text (Chatzi et al., 2024; Niu et al., 2024; Gu et al., 2024; Zhou et al., 2024), images (Zhang et al., 2023b; Gao et al., 2023; Ravi et al., 2024), and even molecular structures (Gong et al., 2024; Dauparas et al., 2022; Zhang et al., 2024). Their outputs are expected to preserve the global integrity of the input while making precise local edits to satisfy the desired condition. Analogous to these models’ consideration of spatial context to edit images (Zhang et al., 2023b; Gao et al., 2023) and protein pockets (Dauparas et al., 2022; Zhang et al., 2024) via in-painting, our work leverages temporal context to perform precise editing on sequences.

Controllable text generation (CTG) approaches, designed specifically to edit natural language sequences, have been extensively studied (Zhang et al., 2023a). They excel in *immediate sequence editing*: predicting the next token or readout in the sequence under a counterfactual condition (Niu et al., 2024; Gu et al., 2024; Zhou et al., 2024; Chatzi et al., 2024; Zhang et al., 2023a; Bhattacharjee et al., 2024). For example, if asked to predict the next word in the sentence “Once upon a time, there lived a boy” under the counterfactual condition that the genre is horror, a CTG model may respond with “alone” to convey vulnerability and loneliness. However, CTG models are unable to perform *delayed sequence editing*: predicting a counterfactual trajectory at a future time step while maintaining causal consistency. For example, if asked to predict what would happen to the boy if he took a trip to New York City as an adult, a CTG model would struggle due to the multitude of possible answers. In the existing paradigm of CTG models, they cannot effectively utilize the given context to skip ahead to the future; instead, CTG models would need to be run repeatedly to fill in the temporal gap without any guarantee of ever satisfying the desired condition. As a result, CTG models are insufficient for other types of sequences for which both immediate and delayed sequence editing are necessary, such as cellular reprogramming and patient immune dynamics.

There exist two controllable time series generation approaches (Jing et al., 2024; Bao et al., 2024), which utilize diffusion modeling to generate counterfactual time series. However, they are limited to univariate sequences and assume that the entire input sequence is affected (Jing et al., 2024; Bao et al., 2024). These methods are thus insufficient in settings where edits are only allowed after time *t* (i.e., cannot change historical data) and affect only certain sequences (i.e., preserve unaffected co-occurring sequences). In other words, they are unable to make precise local edits while preserving global causal consistency.

### Present work.

**Figure 1.**
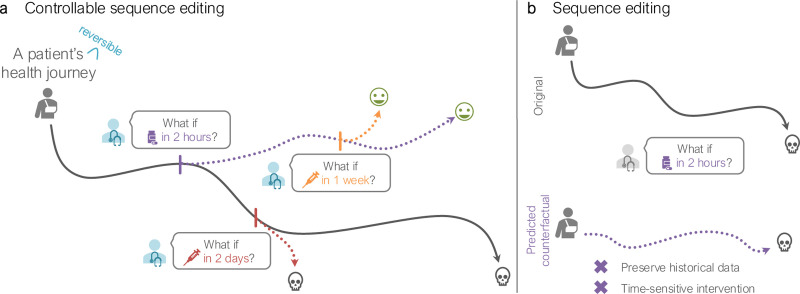
Illustrative comparison of (**a**) Clef’s controllable sequence editing and (**b**) existing sequence editing approaches. Unlike existing methods, controllable sequence editing generates counterfactual sequences (dotted lines) while preserving historical data to model the immediate or delayed effects of interventions.

We tackle the gaps in controllable sequence editing to enable *temporally localized modifications at any time step while ensuring the adherence to temporal causality and the consistency of intrinsic dependencies within and across sequences* (Figure 1). Controllable sequence editing is a particularly complex task because it necessitates learning both the temporal dynamics in the sequences and the relationships between the desired condition and the sequences. The latter is most often unknown, hindering the model’s ability to determine (1) which sequences are affected by the condition, and (2) when and how the sequences are affected based on the historical data and the guiding condition. These challenges prevent sequence editing approaches from generating precise and context-specific edits while preserving temporal and structural constraints. Further, the resulting condition-guided counterfactual sequences may not resemble the distribution of observed sequences.

We develop Clef (ControLlable sequence Editing for counterFactual generation), a controllable sequence editing approach for instance-wise counterfactual generation. Clef learns temporal concepts that represent the trajectories of the sequences to enable accurate counterfactual generation guided by a given condition. We show that the learned temporal concepts help preserve temporal and structural constraints in the generated outputs. By design, Clef is flexible with any type of sequential data encoder. We demonstrate through comprehensive experiments on four novel benchmark datasets in cellular reprogramming and patient immune dynamics that Clef outperforms state-of-the-art models by up to 36.01% and 65.71% (MAE) on immediate and delayed sequence editing, respectively. We also show that any pretrained sequence encoder can gain controllable sequence editing capabilities when finetuned with Clef. Moreover, Clef outperforms baselines in zero-shot counterfactual generation of cellular trajectories by up to 14.45% and 63.19% (MAE) on immediate and delayed sequence editing, respectively. Further, precise edits via user interaction can be performed directly on Clef’s learned concepts. We demonstrate through real-world case studies that Clef, given precise edits on specific temporal concepts, can generate realistic “healthy” counterfactual trajectories for patients originally with type 1 diabetes mellitus.

### Our contributions are threefold.

(1) We formalize controllable sequence editing for temporally localized modification of biomedical sequences at any time step given a desired condition while ensuring temporal causality. (2) Clef is a novel controllable sequence editing model for counterfactual generation. (3) We release four datasets on cellular reprogramming and patient immune dynamics, and demonstrate the efficacy of Clef on immediate and delayed sequence editing on cellular and patient trajectories.

## 2. Related work

### Sequence editing.

The sequence editing task has been defined in language and time series modeling via different terms, but share a core idea: Given a sequence and an alternative condition (e.g., sentiment, attribute), generate a counterfactual sequence with the desired properties. Counterfactual sequence generation is an autoregressive process in language (Chatzi et al., 2024) but a diffusion process in time series (Jing et al., 2024; Bao et al., 2024). Prompting is often used to guide the generation of a sequence, both textual and temporal, with a desired condition (Zhang et al., 2023a; Bhattacharjee et al., 2024; Jing et al., 2024; Bao et al., 2024). However, existing approaches are unable to generate counterfactual multivariate sequences, preserve relevant historical data, and ensure time-sensitive interventions. Temporal sequence editing approaches assume that sequences are univariate and conditions affect the entire sequence (Jing et al., 2024; Bao et al., 2024). While incorporating a structural causal model for token sampling can help preserve certain attributes during counterfactual text generation, the counterfactual statements may be inconsistent with real-world causal models (Chatzi et al., 2024).

### Concept-based learning.

Concepts can be thought of as abstract atomic ideas or concrete tokens of text or images (The et al., 2024; Lai et al., 2024). Concept-based learning has been used to explain (e.g., predict the concepts observed in the sample) or transform black-box models into more explainable models (e.g., allow users to intervene on learned concepts) (Koh et al., 2020; Shin et al., 2023; Ismail et al., 2024; Lai et al., 2024; Laguna et al., 2024; van Sprang et al., 2024). While concepts have been used in sequence generation, they have not yet been used for conditional generation. The adoption of concept-based learning for counterfactual prediction is limited to image classification, where concepts are intervened on during training to simultaneously learn the label and explanation (Dominici et al., 2024). Further, there is a consistent and widely accepted trade-off between accuracy and interpretability in concept-based models.

### Leveraging trajectories as inductive biases.

Understanding sequential data as trajectories (e.g., increasing, decreasing, constant) is more natural for human interpretation than individual values (Kacprzyk et al., 2024). Many modeling approaches on temporal data extract dynamic motifs as inductive biases to improve their interpretability (Kacprzyk et al., 2024; Goswami et al., 2024; Cao et al., 2024). Such temporal patterns can be used for prompting large pretrained models to perform time series forecasting (Cao et al., 2024), suggesting that trajectories can capture more universal and transferrable insights about the temporal dynamics in time series data. Trajectories have yet to be adopted for counterfactual sequence generation.

## 3. Clef

Clef manipulates sequences based on user-specified conditions and temporal coordinates to address “what if” questions. Given a sequence, a forecast time step, and a counterfactual condition, Clef modifies only the relevant portions of the sequence while preserving unaffected elements, ensuring causal consistency. For example, based on a patient’s historical lab test results, Clef generates a future lab test trajectory conditioned on a given treatment (Figure 1a). Architecturally, Clef has four key components: (i) a sequence encoder *F* that extracts temporal features from historical sequence data, (ii) a condition adapter H that maps counterfactual conditions to latent representations, (iii) a concept encoder E that learns temporal concepts, representing trajectory patterns over time, and (iv) a concept decoder G that applies these concepts to generate counterfactual sequences.

### 3.1. Problem definition

#### Definition 3.1 (Sequence editing).

**Figure 2.**
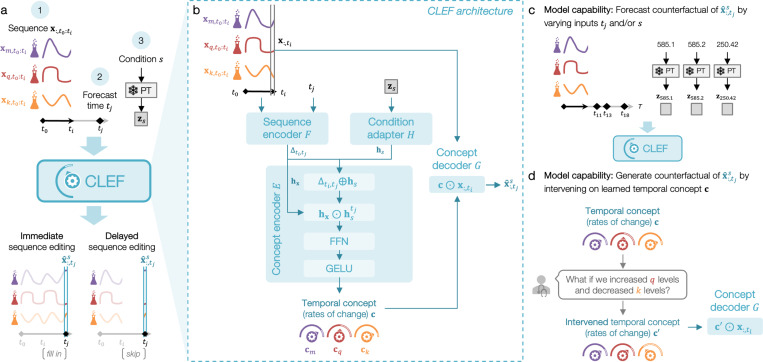
Overview of Clef’s architecture and capabilities. (**a**) Given an input sequence, forecast time, and condition embedding from a frozen pretrained (PT) embedding model, Clef generates a counterfactual sequence via immediate or delayed sequence editing. (**b**) Clef is composed of a sequence encoder, condition adapter, concept encoder, and concept decoder. Clef has two key capabilities: (**c**) forecasting counterfactual sequences at any time in the future and under any condition (e.g., medical codes), and (**d**) generating counterfactual sequences by intervening on Clef’s learned temporal concepts.

Sequence editing is the local sample-level modification of sequence x to generate a counterfactual sequence ${\hat{\text{x}}}_{:,t_{j}}$ under a given condition s at a specific time $t_{j}$. There are two types of controllable sequence editing: immediate and delayed (Figure 2a).

- **Immediate sequence editing:** Given sequence ${\text{x}}_{:,t_{0}:t_{i}}$ and condition s to occur at time $t_{i+1}$, forecast ${\hat{\text{x}}}_{:,t_{i+1}}$.
- **Delayed sequence editing:** Given sequence ${\text{x}}_{:,t_{0}:t_{i}}$ and condition s to occur at time $t_{j}\geq t_{i+1}$, forecast ${\hat{\text{x}}}_{:,t_{j}}$.

Example scenarios for immediate sequence editing include: *What if we perturb the cells now?* and *What if we perform surgery on the patient today?* (Section 5.1). In contrast, delayed sequence editing applies to questions such as: *What if we perturb the cells in ten days?* and *What if we perform surgery on the patient next year?* (Section 5.2).

#### Definition 3.2 (Temporal concept).

A temporal concept c for sequence ${\text{x}}_{:,t_{j}:t_{k}}$ is defined by $\text{c}={\text{x}}_{:,t_{k}}/{\text{x}}_{:,t_{j}}$ for time steps $t_{j}$ and $t_{k}$ where $t_{k}>t_{j}$. It can be interpreted as the trajectory (or rate of change of each variable in the sequence) between any pair of time steps.

#### Definition 3.3 (Controllable sequence editing).

Concept encoder E and decoder G are able to leverage temporal concepts c to perform controllable sequence editing on dataset 𝒟 if the following are satisfied.

- Condition s on ${\text{x}}_{:,t_{0}:t_{i}}$ at time step $t_{j}$ learns c that accurately forecasts ${\hat{\text{x}}}_{:,t_{j}}^{s}$ such that ${\hat{\text{x}}}_{:,t_{j}}^{s}≃{\text{x}}_{:,t_{j}}^{s}$.
- Counterfactual condition a≠s on $x_{:,t_{0}:t_{i}}$ at $t_{j}$ learns $c^{\prime }\neq c$ that forecasts ${\hat{\text{x}}}_{:,t_{j}}^{a}$ such that ${\hat{\text{x}}}_{:,t_{j}}^{a}\neq {\hat{\text{x}}}_{:,t_{j}}^{s}$ and, if known, ${\hat{\text{x}}}_{:,t_{j}}^{a}≃{\text{x}}_{:,t_{j}}^{a}$.

#### Problem Statement 3.1 (Clef).

Given a sequence encoder F, condition adapter H, concept encoder E, and concept decoder G trained on a longitudinal dataset 𝒟, Clef learns temporal concept $\text{c}=E\left(F\left({\text{x}}_{:,t_{0}:t_{i}},t_{j}\right),H(s)\right)$ to forecast ${\hat{\text{x}}}_{:,t_{j}}^{s}=G\left({\text{x}}_{:,t_{i}},\text{c}\right)$ for any sequence ${\text{x}}_{:,t_{0}:t_{i}}\in 𝒟$, future time step $t_{j}>t_{i}$, and condition s.

### 3.2. Clef model

The input to Clef are a multivariate sequence ${\text{X}}_{:,t_{0}:t_{i}}$ with V measured variables, and a condition s and time $t_{j}>t_{i}$ for which to forecast ${\hat{\text{x}}}_{:,t_{j}}^{s}$. Clef consists of four major components: a sequence encoder F, a condition adapter H, a concept encoder E, and a concept decoder G.

#### Sequence encoder F.

The sequence encoder F extracts features from ${\text{x}}_{:,t_{0}:t_{i}}$ such that ${\text{h}}_{\text{x}}=F({\text{x}}_{:,t_{0}:t_{i}})$. Any encoder, including a pretrained multivariate foundation model, can be used. The time encoder in F generates a time positional embedding ${\text{h}}_{t}$ for any t via element-wise summation of the year (sinusoidal), month, date, and hour embeddings. It is additionally used to compute the time delta embedding ${\text{Δ}}_{t_{i},t_{j}}={\text{h}}_{t_{j}}-{\text{h}}_{t_{i}}$ for the concept encoder E.

#### Condition adapter H.

The embedding ${\text{Z}}_{s}$ corresponding to the input condition s is retrieved from a frozen pretrained embedding model (denoted as PT in Figure 2a). The condition adapter H projects ${\text{Z}}_{s}$ into hidden representation ${\text{h}}_{s}=H\left({\text{z}}_{s}\right)$.

#### Concept encoder E.

Given the hidden representations generated by sequence encoder F and condition adapter H, concept encoder E learns temporal concept $\text{c}=E\left({\text{h}}_{\text{x}},{\text{Δ}}_{t_{i},t_{j}},{\text{h}}_{s}\right)$. First, the time delta embedding ${\text{Δ}}_{t_{i},t_{j}}$ is combined via summation with the condition embedding ${\text{h}}_{s}$ to generate a time- and condition-specific embedding ${\text{h}}_{s}^{t_{j}}={\text{Δ}}_{t_{i},t_{j}}⊕{\text{h}}_{s}$. Temporal concept c is learned via an element-wise multiplication of ${\text{h}}_{\text{x}}$ and ${\text{h}}_{s}^{t_{j}}$, an optional linear projection using a feedforward neural network (FNN), and a GELU activation to approximate the trajectory between $t_{i}$ and $t_{j}$

(1) $$\text{c}=\text{GELU}\left(\text{FFN}\left({\text{h}}_{\text{x}}⊙{\text{h}}_{s}^{t_{j}}\right)\right)$$

#### Concept decoder G.

The concept G forecasts ${\hat{\text{x}}}_{:,t_{j}}^{s}$ by performing element-wise multiplication of the latest time $t_{i}$ of the input sequence ${\text{x}}_{:,t_{0}:t_{i}}$ (denoted as ${\text{x}}_{:,t_{i}}$) and the learned concept c

(2) $${\hat{\text{x}}}_{:,t_{j}}^{s}=\text{c}⊙{\text{x}}_{:,t_{i}}$$

#### Objective function 𝓛.

The sequence editing objective function 𝓛 quantifies the reconstruction error of the predicted ${\hat{\text{x}}}_{:,t_{j}}^{s}$ and the ground truth ${\text{x}}_{:,t_{j}}^{s}$. Here, we use Huber loss

(3) $$𝓛\left({\text{x}}_{:,t_{j}}^{s},{\hat{\text{x}}}_{:,t_{j}}^{s}\right)=\{\begin{array}{ll} 0.5{\text{a}}^{2}, & \text{if}\;|\text{a}|\leq \delta \\ \delta (|\text{a}|-0.5\delta ), & \text{otherwise} \end{array}$$

where $\text{a}={\text{x}}_{:,t_{j}}^{s}-{\hat{\text{x}}}_{:,t_{j}}^{s}$.

## 4. Experimental setup

### 4.1. Datasets

**Figure 3.**
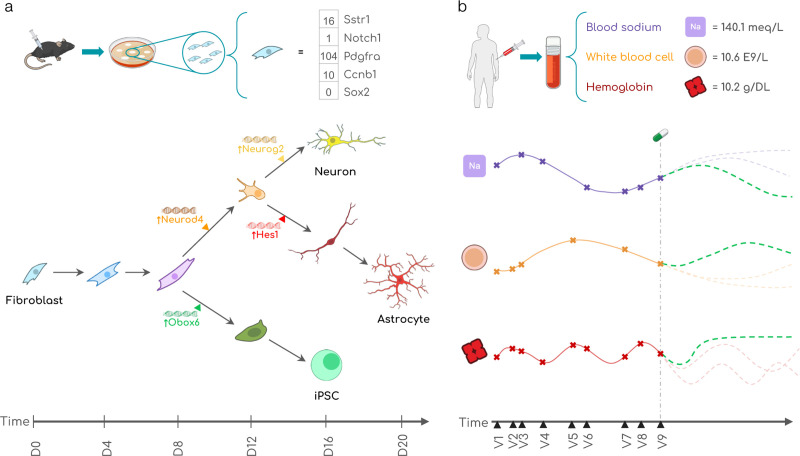
Clef is evaluated on two real-world domains involving multivariate trajectories: (**a**) cellular development and (**b**) patient health. (**a**) To study cellular development, fibroblast cells derived from mice can be artificially reprogrammed into various other cell states *in vitro*. A cell’s state is defined by its gene expression. Throughout reprogramming, a cell activates transcription factor (TF) genes at different time points to change its gene expression, thereby influencing its developmental trajectory. In this illustration, a mouse fibroblast is being reprogrammed over the span of 20 days (D0-D20); color and shape represent cell state. On day 8, if the cell activates the Obox6 TF, the cell is on the path toward becoming an induced pluripotent stem cell (iPSC); whereas if it activates the Neurod4 TF, it is on the path toward becoming a neuron or astrocyte. **(b)** The health of a human patient is often monitored through lab tests (e.g. blood sodium level, white blood cell count). The history of lab results across multiple patient visits (V1-V9) as well as candidate clinical interventions (e.g., medication) can be used to infer the most likely future trajectory of the patient’s health. Illustrations from NIAID NIH BIOART Source (see References).

Clef is evaluated on datasets and tasks in the biological and medical domains: cellular reprogramming experiments (Figure 3a) and patient routine laboratory tests (Figure 3b).

#### Cellular developmental trajectories.

We introduce a novel benchmarking dataset, WOT. It is constructed using the Waddington-OT model, which simulates single-cell transcriptomic profiles of developmental time courses for individual cells (Schiebinger et al., 2019) (Figure 3a; Table 1). We also construct a paired counterfactual benchmarking dataset, WOT-CF (Table 1). We obtain condition embeddings of the activated transcription factors from ESM-2 (Lin et al., 2022). Refer to Appendix A.1 for further details.

#### Patient lab test trajectories.

We construct two real-world patient datasets of routine laboratory tests from eICU (Pollard et al., 2018) and MIMIC-IV (Johnson et al., 2024a; 2023; Goldberger et al., 2000) (Figure 3b; Table 1). In addition to a random split, we construct data splits with different levels of train/test split similarities using SPECTRA (Ektefaie et al., 2024) to evaluate model generalizability (Appendix Figure 10). For condition embeddings, we lever-age pretrained embeddings of clinical codes from a clinical knowledge graph that integrates six existing databases of clinical vocabularies used in electronic health records (Johnson et al., 2024b). Refer to Appendix A.2 for more details.

### 4.2. Setup

#### Metrics.

We use standard metrics (MAE, RMSE, and *R*^2^) to quantify sequence editing performance.

#### Baselines.

We evaluate Clef against a traditional multivariate time series algorithm, Vector Autoregression (VAR) model (Lütkepohl, 2005). As Clef can leverage any type of sequence encoder, we benchmark against the state-of-the-art condition-guided counterfactual sequence generation setup with different sequential data encoders: Transformer (Waswani et al., 2017; Narasimhan et al., 2024; Jing et al., 2024; Zhang et al., 2023a) and xLSTM (Beck et al., 2024). We further evaluate Clef against a state-of-the-art time series foundation model, MOMENT (Goswami et al., 2024); specifically, we finetune an adapter for the 1024-dimensional embeddings generated by the frozen MOMENT-1-large embedding model.

#### Ablations.

To investigate the effectiveness of the learned temporal concepts, we evaluate against an ablated model, SimpleLinear, in which temporal concepts are simply all ones; in other words, temporal concepts are not learned nor meaningful. This ablation is inspired by traditional linear models that excel when ${\text{x}}_{t_{j}}≃{\text{x}}_{t_{i}}$ (Toner & Darlow, 2024; Ahlmann-Eltze et al., 2024). We also evaluate different versions of Clef with and without an FFN layer in the concept encoder *E* (Appendix C).

#### Implementation details.

Models are trained on a single NVIDIA A100 or H100 GPU. All models have comparable number of parameters as their Clef-based counterparts. Refer to Appendix B for details and hyperparameter selection.

## 5. Results

We evaluate Clef’s performance on controllable sequence editing across multiple datasets and tasks. We aim to answer the following research questions. **R1:** How well does Clef perform in immediate sequence editing? **R2:** How well does Clef perform in delayed sequence editing? **R3:** How does Clef generalize to unseen/new sequences? **R4:** Can Clef perform zero-shot counterfactual generation? **R5:** How can Clef be leveraged for real-world counterfactual patient trajectory simulations? We establish that Clef outperforms state-of-the-art baselines in sequence editing, demonstrating both immediate and delayed sequence editing capabilities, strong generalizability, and real-world applicability.

### 5.1. R1: Immediate sequence editing

Immediate sequence editing involves forecasting the next time step of a sequence under a counterfactual condition. This is useful in settings where interventions take effect instantaneously, such as introducing a genetic perturbation in cellular systems or administering a drug to a patient (Definition 3.1). Example counterfactual scenarios in which immediate sequence editing is applicable are: *What if we treat the cells with the candidate drug now?* and *What if we perform surgery on the patient today?*

**Figure 4.**
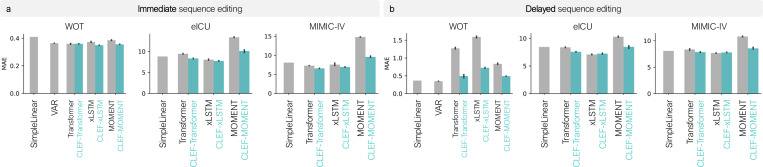
Benchmarking the performance of Clef, baselines, and ablation models on (**a**) immediate and (**b**) delayed sequence editing. The models are trained using a standard cell- or patient-centric random split. Not shown for visualization purposes are the performances of VAR models on eICU and MIMIC-IV datasets: on immediate sequence editing, MAE for eICU and MIMIC-IV are 55982.74 and 886.05, respectively; on delayed sequence editing, MAE for eICU and MIMIC-IV are 3.02 × 10^39^ and 8.62 × 10^23^, respectively.

Clef models consistently outperform baseline models across all datasets (Figure 4a; Appendix Figures 8–9). The SimpleLinear baseline, which assumes minimal temporal changes, performs comparably in some cases, but Clef outperforms it on datasets where short-term dynamics are more complex. On WOT, all Clef models outperform or perform comparably to the time series forecasting model, VAR. This is particularly exciting given recent findings that linear models can achieve competitive or better forecasting performance than neural network models (Toner & Darlow, 2024; Ahlmann-Eltze et al., 2024). These results highlight Clef’s ability to accurately modify trajectories at the right points while preserving unaffected portions of the sequence, an advantage in counterfactual reasoning.

Regardless of the sequence encoder used with Clef, these models tend to outperform or perform comparably to non-Clef models (Figure 4a). However, the performance of Clef can be affected by the ability of the sequence encoder to capture the temporal dynamics of the input sequences. For instance, models with the MOMENT encoder yield the highest MAE in all three datasets, with and without help from Clef (Figure 4a). Nevertheless, Clef models with the MOMENT encoder reduce the MAE of non-Clef models.

### 5.2. R2: Delayed sequence editing

Delayed sequence editing requires forecasting a counter-factual trajectory at a future time step while maintaining causal consistency. This task is challenging, as small errors can compound over longer horizons. Example scenarios in which delayed sequence editing is applicable are: *What if we treat the cells with the candidate drug in ten days?* and *What if we perform the surgery on the patient next year?*

Clef outperforms or performs competitively against SimpleLinear and VAR on the patient datasets, eICU and MIMIC-IV (Figure 4b; Appendix Figures 8–9). Clef-transformer and Clef-xLSTM achieve lower MAE than SimpleLinear, whereas non-Clef transformer and MOMENT baselines perform comparably or worse. As in immediate sequence editing, models using MOMENT as the sequence encoder (i.e., using temporal concepts with the MOMENT sequence encoder) yield the highest MAE. However, incorporating Clef with MOMENT reduces the MAE to levels comparable to SimpleLinear and VAR.

On WOT, SimpleLinear and VAR outperform neural network models in delayed sequence editing (Figure 4b). This suggests that cellular developmental trajectories exhibit small and possibly noisy changes at each time step, favoring linear models (Ahlmann-Eltze et al., 2024; Toner & Darlow, 2024). Additionally, given the relatively small number of training trajectories compared to the high-dimensional state space, nonlinear models may overfit to noise more readily than linear models. Nevertheless, Clef significantly reduces the MAE of non-Clef models, demonstrating its effectiveness as a regularizer that mitigates short-term noise while preserving long-term trends.

### 5.3. R3: Generalization to new patient trajectories

We assess the ability of Clef models to generalize to new patient sequences. To evaluate robustness, we use the SPECTRA approach (Ektefaie et al., 2024) to create challenging data splits where the test sets have minimal similarity to the training data (Appendix A.2).

**Figure 5.**
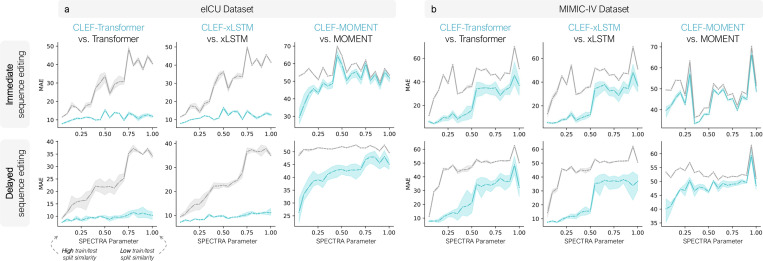
Generalizability of Clef on (**a**) eICU and (**b**) MIMIC-IV patient datasets in immediate and delayed sequence editing. As the SPECTRA parameter increases, the train/test split similarity decreases (Appendix Figure 10). The area under the spectral performance curve (AUSPC) evaluation is in Appendix Table 2.

Across both the eICU and MIMIC-IV patient datasets, Clef models exhibit stronger generalization than non-Clef models (Figure 5; Appendix Figures 11–12 and Table 2). For immediate and delayed sequence editing on eICU, Clef-transformer and Clef-xLSTM maintain stable and strong performance even as train/test divergence increases. In contrast, their non-Clef counterparts degrade significantly. Although baseline MOMENT models show relatively stable performance across train/test splits in delayed sequence editing, they generalize poorly compared to Clef-MOMENT models. Despite similar performance between xLSTM and Clef-xLSTM in delayed sequence editing on both patient datasets (Figure 4b), Clef-xLSTM demonstrates superior generalizability (Figure 5b), highlighting the effectiveness of Clef in adapting to unseen data distributions.

### 5.4. R4: Zero-shot counterfactual generation of cellular trajectories

**Figure 6.**
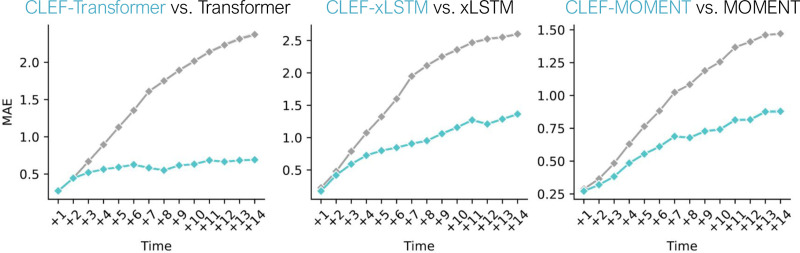
Zero-shot counterfactual generation of cellular developmental trajectories. Shown are the MAE of predictions at each time step for counterfactual sequences of length 23 (the most common sequence length in the dataset) starting at time step 10 (the earliest divergence time step of a counterfactual trajectory).

In addition to evaluating Clef’s generalizability to new patient lab test trajectories, we assess on zero-shot counterfactual generation for cellular trajectories (Figure 6; Appendix Figure 13). Using the Waddington-OT model, we generate sequences that remain consistent until a specified divergence time step, where an alternative condition—such as the activation of a different transcription factor—introduces a shift (Appendix A.1). This process yields 1,273 pairs of “original” and “counterfactual” trajectories, totaling 2,546 individual sequences (Table 1). Models are trained on the “original” trajectories and evaluated on the “counterfactual” trajectories in a zero-shot setting.

Clef-based models consistently outperform non-Clef models in both immediate and delayed sequence editing (Appendix Figure 13). To more closely analyze delayed sequence editing performance, we examine the predictions for cellular trajectories of length 23, the most common sequence length in the dataset (Figure 6). Since $t_{i}=10$ is the earliest divergence time step, we provide the first nine time steps ${\text{x}}_{:,0:9}$, the counterfactual condition, and $t_{j}\in [10,23]$ to the model. Comparing the generated and ground truth counterfactual sequences, we find that Clef significantly outperforms non-Clef models after time step 10, which is when the trajectories begin to diverge (Figure 6).

### 5.5. R5: Case studies using real-world patient datasets

We evaluate Clef’s ability to simulate counterfactual patient trajectories through temporal concept intervention. We conduct case studies on two independent cohorts of patients with type 1 diabetes mellitus (T1D), a chronic autoimmune disease in which the immune system attacks insulin-producing cells in the pancreas (Quattrin et al., 2023).

Unlike counterfactual generation methods that rely on condition tokens to guide generation (Narasimhan et al., 2024; Jing et al., 2024; Zhang et al., 2023a), Clef allows *direct edits to the generated outputs* to produce counterfactual sequences. This capability is particularly valuable when condition tokens are insufficient, such as when prescribing medication dosage. Instead of relying on predefined conditions, Clef can precisely modify the values of specific lab tests to explore their longitudinal effects.

#### Setup.

For an individual patient, we intervene on the temporal concepts corresponding to specific lab tests to simulate the “reversal” or “worsening” of symptoms, thereby generating “healthier” or “more severe” trajectories, respectively. Formally, given temporal concept c learned from ${\text{X}}_{:,t_{0}:t_{i}}$ and an optional condition s, we modify ${\text{c}}^{I}\neq \text{c}$ such that at least one element satisfies ${\text{c}}_{k}\neq {\text{c}}_{k}^{I}$.

From the eICU and MIMIC-IV datasets, we construct two independent cohorts of T1D patients and matched healthy individuals (Appendix A.2). The eICU-T1D dataset contains 59 T1D patients and 579 matched healthy controls, while MIMIC-IV-T1D includes 25 T1D patients and 226 matched healthy controls.

To generate counterfactual sequences, we modify specific values in temporal concept **c**, such as glucose levels, and allow Clef to simulate future trajectories of length *T* = 10. We then compare these counterfactual trajectories (i.e., Clef-generated patients) against observed sequences from matched healthy individuals, other healthy individuals, and other T1D patients. Our hypothesis is that clinically meaningful edits will produce “healthier” (i.e., more similar to healthy patients) or “sicker” (i.e., more similar to other T1D patients) trajectories.

#### Results.

**Figure 7.**
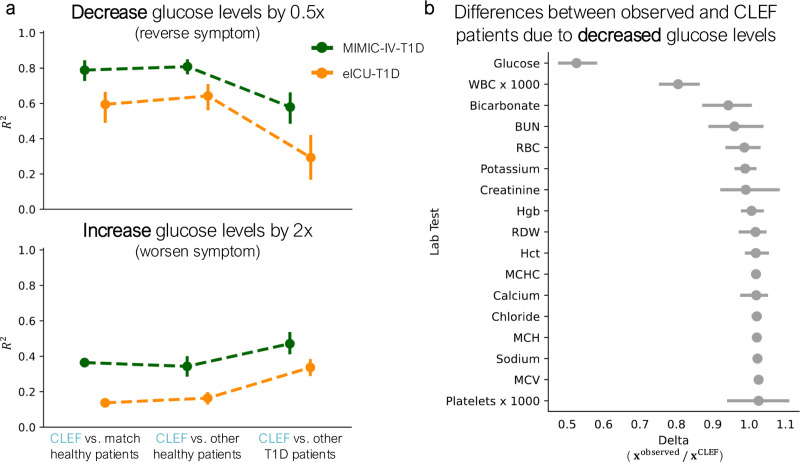
Clef-generated counterfactual patients via intervention on temporal concepts. We intervene on Clef to (**a**) halve (top) or double (bottom) a T1D patient’s glucose levels to infer a “healthier” or “sicker” counterfactual patient, respectively. (**b**) Observed and Clef patients from the eICU-T1D cohort are compared to quantify the differences between their lab test trajectories (indirect effects) as a result of the intervention to halve T1D patients’ glucose levels.

First, we modify Clef’s concepts to reduce glucose levels by half, aligning them closer to normal physiological ranges. The resulting counterfactual patient trajectories exhibit higher *R*^2^ similarity with both matched and other healthy individuals compared to other T1D patients (Figure 7a). This suggests that Clef effectively generates counterfactual trajectories indicative of a healthier state.

Next, we simulate a worsening condition by doubling glucose levels. The resulting counterfactual trajectories generated by Clef show higher *R*^2^ similarity with other T1D patients than with healthy individuals (Figure 7a), as would be expected based on clinical evidence.

Beyond direct interventions, we examine indirect changes in Clef-generated patients’ lab values resulting from glucose modifications. In both eICU-T1D and MIMIC-IV-T1D cohorts, lowering glucose also leads to a reduction in white blood cell (WBC) count (Figure 7b; Appendix Figure 14a). This aligns with clinical knowledge, as T1D is an autoimmune disorder where immune activity, including WBC levels, plays a critical role (Quattrin et al., 2023). Further, when we intervene on Clef to reduce WBC levels instead of glucose, we observe a concurrent drop in glucose across both cohorts (Appendix Figure 14b,c), reinforcing the interconnected nature of these physiological markers.

Finally, we demonstrate that modifying multiple lab tests simultaneously can produce compounding effects. When we intervene on Clef to reduce both glucose and WBC levels, the resulting Clef-generated patients resemble healthy individuals even more closely than other T1D patients (Appendix Figure 14d). This finding suggests that Clef can integrate multiple simultaneous edits, capturing their joint impact on a patient’s future state.

## 6. Conclusion

In this work, we formalize controllable sequence editing for counterfactual generation on biomedical sequences, and demonstrate that Clef outperforms state-of-the-art counterfactual sequence generation models in immediate and delayed sequence editing. Clef also has stronger generalizability to new sequences, and performs significantly better than state-of-the-art models in zero-shot counterfactual generation. Further, we show that interventions directly on Clef’s temporal concepts can generate counterfactual patients such that their trajectories are shifted toward healthier outcomes. This capability has the potential to help discover clinical interventions that could alleviate a patient’s symptoms. While this work focuses on cellular and patient trajectories, Clef can be readily extended to perform sequence editing in other domains.

### Limitations.

There are two key limitations of Clef. Firstly, we define temporal concepts such that each element represents a unique measured variable in the sequence (e.g., gene expression, lab test). Instead, it may be beneficial to learn higher-order relationships between the measured variables or across time as abstract hierarchical concepts (The et al., 2024; Kacprzyk et al., 2024). Secondly, while Clef is able to generate counterfactual sequences for any condition, including those it may not have seen during training, Clef could potentially improve with additional guidance from a real-world causal model for the system or domain of interest (Chatzi et al., 2024). Since defining such a real-world causal graph is a major challenge, one promising future direction could be to enable user interventions, such as those performed in our T1D case studies, to finetune Clef.

### Broader Impact

By introducing a flexible and interpretable approach to counterfactual sequence generation, Clef bridges the gap between language model-style conditional generation and structured, time-sensitive sequence editing, with implications for decision support in medical and scientific applications. Like all generative AI models, Clef (and its derivatives) should be used solely for the benefit of society. In this study, we demonstrate that Clef can generate alternative cellular trajectories and simulate the reversal or progression of symptoms to model healthier or sicker patient outcomes. However, this work (and any derivatives) should never be used to induce harmful cellular states (e.g., activating transcription factors to drive a cell toward a pathological state) or negatively impact patient care (e.g., neglecting necessary clinical interventions or recommending harmful treatments). Our goal is to help researchers understand the underlying mechanisms of disease to improve public health. Any misuse of this work poses risks to patient well-being. Therefore, the ability to intervene on Clef’s generated outputs should be leveraged to assess the model’s robustness and correctness for ethical and responsible use.

## Figures and Tables

**Table 1.** Dataset statistics. We construct three core datasets: WOT (cellular developmental trajectories), eICU (patient lab tests), and MIMIC-IV (patient lab tests). We also construct a paired counterfactual cellular trajectories dataset, WOT-CF. *N* is the number of sequences (i.e., cellular developmental trajectories, patient lab test trajectories), *V* is the number of measured variables (i.e., gene expression, lab test), and *L* is the length of the sequences.

Dataset	*N*	*V*	Mean *L*	Max *L*

WOT	3, 000	1, 480	27.03 ± 6.04	37
WOT-CF	2, 546	1, 480	27.01 ± 5.98	37
eICU	108, 346	17	20.27 ± 25.23	858
MIMIC-IV	156, 310	16	15.56 ± 24.43	949

**Figure 8.**
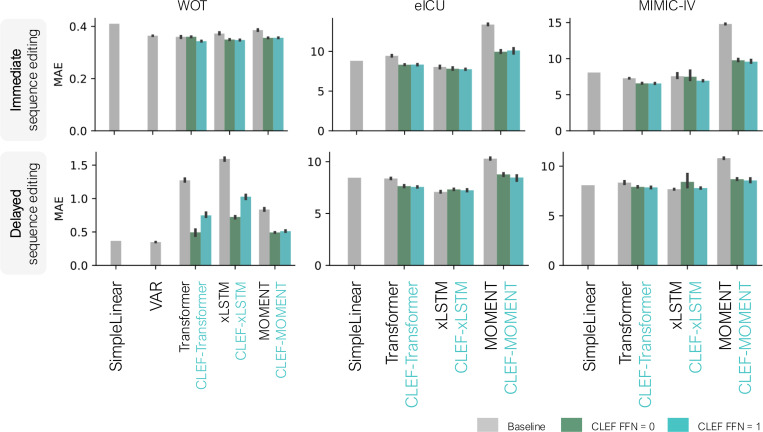
Benchmarking the performance of Clef, baselines, and ablation models on (**a**) immediate and (**b**) delayed sequence editing. Performance is measured by MAE. The models are trained using a standard cell- or patient-centric random split. Not shown for visualization purposes are the performances of VAR models on eICU and MIMIC-IV datasets: on immediate sequence editing, MAE for eICU and MIMIC-IV are 55982.74 and 886.05, respectively; on delayed sequence editing, MAE for eICU and MIMIC-IV are 3.02 × 10^39^ and 8.62 × 10^23^, respectively.

**Figure 9.**
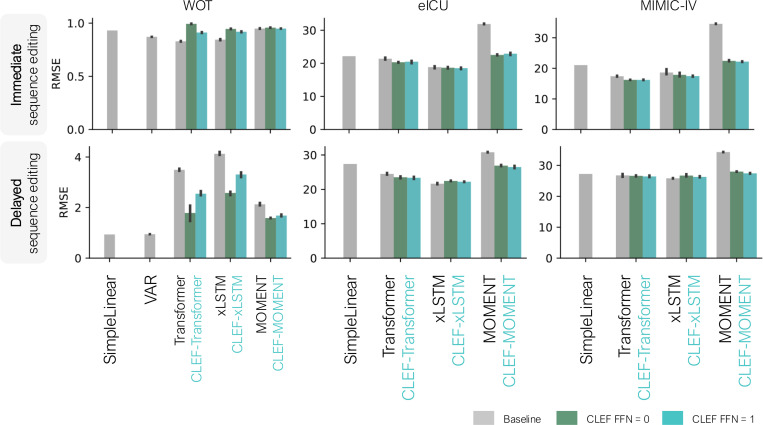
Benchmarking the performance of Clef, baselines, and ablation models on (**a**) immediate and (**b**) delayed sequence editing. Performance is measured by RMSE. The models are trained using a standard cell- or patient-centric random split. Not shown for visualization purposes are the performances of VAR models on eICU and MIMIC-IV datasets: on immediate sequence editing, MAE for eICU and MIMIC-IV are 135003.67 and 1793.23, respectively; on delayed sequence editing, MAE for eICU and MIMIC-IV are 5.84 × 10^39^ and 1.59 × 10^24^, respectively.

**Figure 10.**
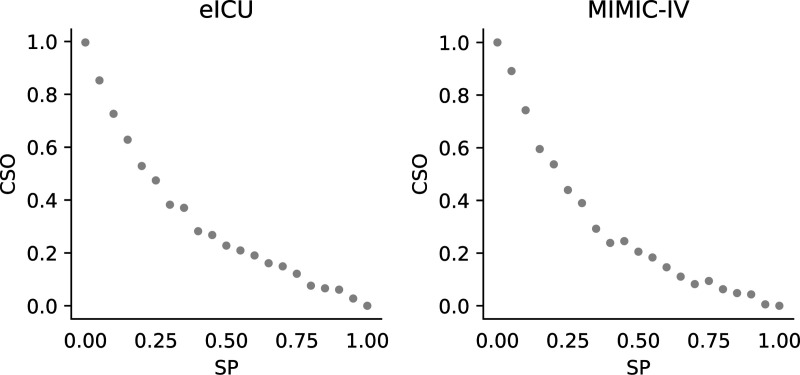
Cross-split overlap (CSO) as a function of SPECTRA parameter (SP) for eICU and MIMIC-IV datasets. CSO is defined as the number of samples in the test set that are similar to at least one sample in the train set. SP is an internal parameter used by SPECTRA to control the CSO of generated data splits. CSO decreases as SP increases.

**Figure 11.**
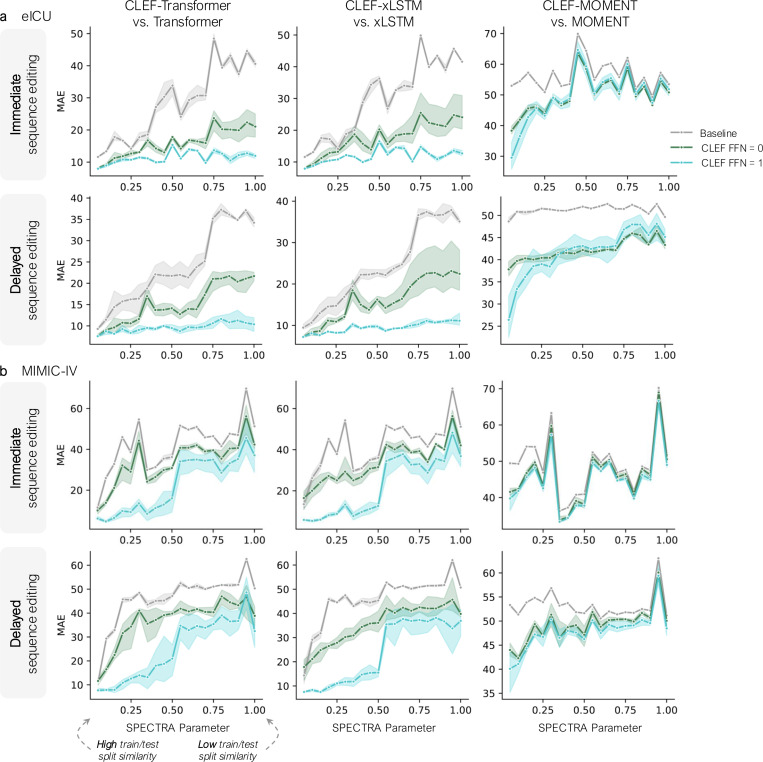
Generalizability of Clef, baselines, and ablation models on **(a)** eICU and **(b)** MIMIC-IV patient datasets in immediate and delayed sequence editing. Performance is measured by MAE. As the SPECTRA parameter increases, the train/test split similarity decreases (Appendix Figure 10). The area under the spectral performance curve (AUSPC) evaluation is in Appendix Table 2.

**Figure 12.**
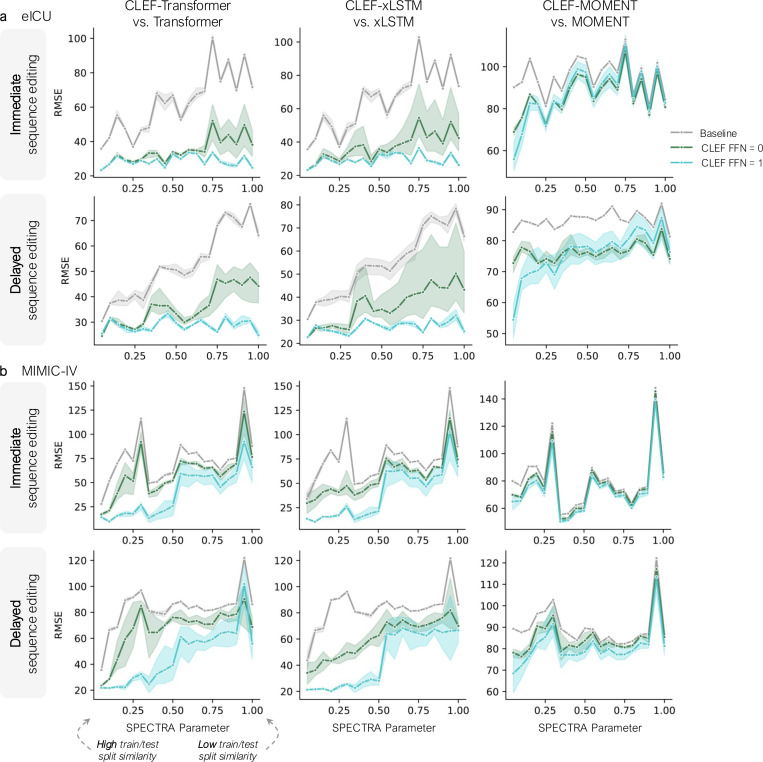
Generalizability of Clef, baselines, and ablation models on **(a)** eICU and **(b)** MIMIC-IV patient datasets in immediate and delayed sequence editing. Performance is measured by RMSE. As the SPECTRA parameter increases, the train/test split similarity decreases (Appendix Figure 10). The area under the spectral performance curve (AUSPC) evaluation is in Appendix Table 2.

**Figure 13.**
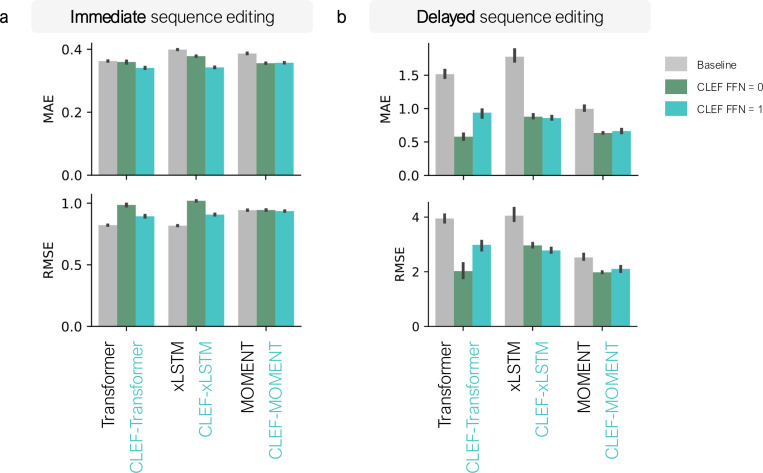
Benchmarking the performance of Clef, baselines, and ablation models on zero-shot (**a**) immediate and (**b**) delayed counter-factual generation of cellular developmental trajectories. Performance is measured by MAE (top row) and RMSE (bottom row).

**Figure 14.**
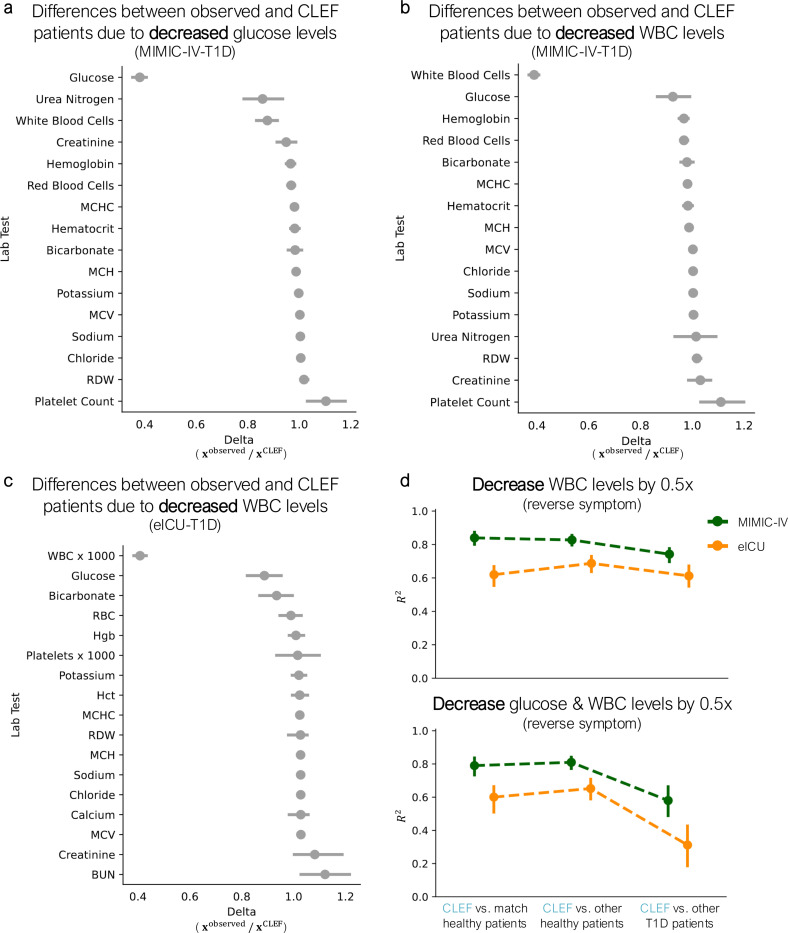
Clef-generated counterfactual patients via intervention on temporal concepts. Observed and Clef patients are compared to quantify the differences between their lab test trajectories as a result of the intervention to halve the (**a**) glucose levels in T1D patients from the MIMIC-IV-T1D cohort, (**b**) white blood cell (WBC) levels in T1D patients from the MIMIC-IV-T1D cohort, and (**c**) WBC levels in T1D patients from the eICU-T1D cohort. (**d**) After intervening on Clef to halve WBC levels, we observe whether the resulting Clef patients’ trajectories are “healthier” or “sicker” compared to other patients in the real-world cohort (top). Further, we investigate whether the intervention effects are compounded when simultaneously reducing glucose and WBC levels by half (bottom).

## A. Data & Experimental Setup

In this section, we provide further details about data construction, data preparation, and experimental setup. We share code and instructions in our GitHub repository to reproduce the experiments in this paper: https://github.com/mims-harvard/CLEF.

### A.1. Cellular Developmental Trajectories

Here, we describe the process of (1) simulating single-cell transcriptomic profiles of developmental time courses for individual cells and (2) preparing these trajectories for modeling.

#### A.1.1. SIMULATING TRAJECTORIES

Cellular reprogramming experiments help elucidate cellular development (Schiebinger et al., 2019). In these experiments, cells are manipulated and allowed to progress for a specific period of time before they undergo RNA sequencing (RNA-seq), and we analyze the resulting RNA-seq data to observe their new cellular profiles (Schiebinger et al., 2019). RNA-seq is a destructive process for the cell, meaning that the same cell cannot be sequenced at two different time points. Computational models are thus necessary to infer the trajectory of a cell.

##### Waddington-OT dataset and model.

Waddington-OT (Schiebinger et al., 2019) is a popular approach to reconstruct the landscape of cellular reprogramming using optimal transport (OT). There are two components in Waddington-OT: (1) *a single-cell RNA-seq (scRNA-seq) dataset* of mouse cells from a reprogramming experiment, and (2) *an OT-based trajectory inference model* fitted on the scRNA-seq dataset. The scRNA-seq dataset consists of 251,203 mouse cells profiled from 37 time points (0.5-day intervals) during an 18-day reprogramming experiment starting from mouse embryonic fibroblasts. The trajectory inference model consists of transport matrices $\pi_{t_{k},t_{k+1}}$ with dimensions N×M that relate all cells ${\text{x}}_{t_{k}}^{1},\ldots ,{\text{x}}_{t_{k}}^{n}$ profiled at time $t_{k}$ to all cells ${\text{x}}_{t_{k+1}}^{1},\ldots ,{\text{x}}_{t_{k+1}}^{m}$ profiled at time $t_{k+1}$. An entry at row i and j of $\pi_{t_{k},t_{k+1}}$ corresponds to the probability ${\text{x}}_{t_{k+1}}^{j}$ is a descendant cell of ${\text{x}}_{t_{k}}^{i}$, as determined using optimal transport (Chizat et al., 2017). Every cell in the scRNA-seq dataset is either pre-labeled as one of the 13 provided cell sets (i.e., induced pluripotent stem, stromal, epithelial, mesenchymal-epithelial transition, trophoblast, spongiotrophoblast, trophoblast progenitor, oligodenrocyte progenitor, neuron, radial glial, spiral artery trophoblast giant, astrocyte, other neural) or unlabeled. We cluster the unlabeled cells using Leiden clustering via scanpy (Wolf et al., 2018) at a resolution of 1, and define the resulting 27 unlabeled clusters as unique cell sets. As a result, each cell in the dataset belongs one and only one cell set.

##### Simulating cell state trajectories.

We define “cell state” as the transcriptomic profile of a cell. Here, a transcriptomic profile is the log-normalized RNA-seq counts of the top 1,479 most highly variable genes. To create a simulated trajectory of cell states for an individual cell undergoing reprogramming, we randomly and uniformly sample a cell profiled at time step $t_{0}$ (Day 0.0) from the Waddington-OT scRNA-seq dataset, and generate via the transport $\pi_{t_{0},t_{1}}$ a probability distribution ${\text{ℙ}}_{t_{1}}$ over possible descendant ${\text{x}}_{t_{1}}^{1},\ldots ,{\text{x}}_{t_{1}}^{m}$ at time step $t_{1}$ (Day 0.5 ). We sample a cell from this distribution, and repeat the process until we reach either Day 18.0 or a terminal state (i.e., neural, stromal, or induced pluripotent stem cell). After generating a trajectory composed of cells from the Waddington-OT scRNA-seq dataset through this process, we retrieve the transcriptomic profile of each cell to ${\text{x}}_{:,t_{0}:t_{T}}$, where T is the length of the trajectory.

##### Inferring conditions.

Inferring conditions. A condition $s_{t_{i}}$ is defined as the activation of a transcription factor (TF) that leads a cell to transition from state ${\text{x}}_{t_{i}}$ to descendant state ${\text{x}}_{t_{i+1}}$. To infer such conditions, we perform differential expression analysis between cells from the same cell set as ${\text{x}}_{t_{i}}$ (i.e., ${\text{x}}^{a}\in A$) and cells from the same cell set ${\text{x}}_{t_{i+1}}$ (i.e ${\text{x}}^{b}\in B$ ). Using the wot.tmap.diff_exp function (via the Waddington-OT library), we identify the top TF that was significantly upregulated in ${\text{x}}^{a}\in A$ compared to ${\text{x}}^{b}\in B$. If no TFs are differentially expressed, then the condition is “None.” We retroactively perform this analysis on all pairs of consecutive cell states in a cell state trajectory ${\text{x}}_{:,t_{0}:t_{T}}$ to obtain the full trajectory containing both cell states and TF conditions: $\tau =\left\{{\text{x}}_{t_{0}},s_{t_{0}},{\text{x}}_{t_{1}},s_{t_{1}},\cdots ,s_{t_{T-1}},{\text{x}}_{t_{T}}\right\}$. In other words, τ represents a simulated trajectory of an individual cell undergoing the reprogramming process. Condition ${\text{z}}_{s}\in {\text{ℝ}}^{5120}$ are obtained from the (frozen) pretrained ESM-2 embedding model (Lin et al., 2022).

##### Generating matched counterfactual trajectories.

We additionally create pairs of matched counterfactual trajectories to evaluate a model’s performance in zero-shot counterfactual generation. Each pair consists of an “original” trajectory $\tau_{og}$ and a “counterfactual” trajectory $\tau_{cf}$. First, we generate $\tau_{og}$ using the Waddington-OT model. Then, given a divergence time step D, the first D time steps $\tau_{og}$ are carried over to $\tau_{cf}$ such that the first D cell states and conditions of $\tau_{og}$ and $\tau_{cf}$ are exactly the same. The remaining states and conditions of $\tau_{cf}$ are sampled independently from $\tau_{og}$, resulting in an alternative future trajectory based on an alternative condition at time step D.

##### Implementation note:

Because Clef learns time embeddings based on the year, month, date, and hour of a given timestamp, we convert the time steps of each cell into timestamps. We set the starting time $t_{0}$ as timestamp 2000/01/01 00:00:00, and add $10\times t_{i}$ hours to the converted timestamp of $t_{i-1}$.

#### A.1.2. Experimental setup

##### Generating data splits.

There are three cell sets (i.e., groups of cells with the same cell state label) that consist of cells from Day 0.0 in our post-clustering version of the Waddington-OT dataset. We refer to these cell sets as “start clusters” because all initial cell states are sampled from one of these cell sets. Since the choice of start cluster can influence the likelihood of a cell’s trajectory reaching certain terminal fates, we split our cellular trajectories into train, validation, and test sets based on their start cluster. This cell-centric data split allows us to evaluate how well a model can generalize to different distributions of trajectories. Start cluster #1 is in the train set, start cluster #3 is in the validation set, and start cluster #2 is in the test set.

##### Zero-shot counterfactual generation.

The data split for zero-shot counterfactual generation is constructed such that the original trajectories $\tau_{og}$ are in the train or validation sets, and the counterfactual trajectories $\tau_{cf}$ are in the test set.

### A.2. Patient Lab Tests

Here, we describe the process of (1) preprocessing electronic health records to extract longitudinal routine lab tests data and (2) preparing these trajectories for modeling.

#### A.2.1. Constructing routine lab test trajectories

We leverage two publicly available medical datasets: eICU (Pollard et al., 2018) and MIMIC-IV (Johnson et al., 2024a; 2023; Goldberger et al., 2000). Both datasets are under the PhysioNet Credentialed Health Data License 1.5.0 (PhysioNet). The retrieval process includes registering as a credentialed user on PhysioNet, completing the CITI “Data or Specimens Only Research” training, and signing the necessary data use agreements.

##### Processing patient datasets.

We process each dataset (i.e., eICU, MIMIC-IV) separately with the following steps. First, we extract the routine lab tests only (annotation available only in MIMIC-IV) and the most commonly ordered lab tests (i.e., lab tests that appear in at least 80% of patients). Next, we keep patients for whom we have at least one of each lab test. If there are multiple measurements of a lab test at the same time step (i.e., year, month, date, hour, minute, and seconds), we take the mean of its values. We extract patients with more than one visit (or time step).

We define patients’ conditions as medical codes, specifically International Classification of Diseases (ICD), of their diagnosis. Both eICU and MIMIC-IV use ICD-9 and ICD-10 codes. We extract the medical codes and their timestamps (multiple medical codes at a single time step is possible). Since the timestamps of diagnostic codes and lab tests are not necessarily the same (and there are fewer entries of diagnostic codes than lab orders), we merge them with a tolerance range of 12 hours (eICU) or two days (MIMIC-IV). We obtain (frozen) condition embeddings ${\text{z}}_{s}\in {\text{ℝ}}^{128}$ (retrieved on December 22, 2024) from an embedding model that has been pretrained on a clinical knowledge graph (Johnson et al., 2024b). The clinical knowledge graph is constructed by integrating six existing databases of clinical vocabularies used in electronic health records: International Classification of Diseases (ICD), Anatomical Therapeutic Chemical (ATC) Classification, Systemized Nomenclature of Medicine - Clinical Terms (SNOMED CT), Current Procedural Terminology (CPT), Logical Observation Identifiers Names and Codes (LOINC), and phecodes (Johnson et al., 2024b).

#### A.2.2. Generating data splits

We generate a standard patient-centric random split for benchmarking model performance, and a series of increasingly challenging data splits via SPECTRA (Ektefaie et al., 2024) to evaluate model generalizability.

##### Constructing data splits to evaluate model generalizability.

SPECTRA (Ektefaie et al., 2024) creates a series of splits with decreasing cross-split overlap or similarity between the train and test sets. By training and testing models on these splits, we can assess model performance as a function of cross-split overlap. SPECTRA refers to this relationship as the spectral performance curve, which provides insight into how well a model generalizes to less similar data. When a new dataset split is encountered, it can be plotted as a point on this curve. The area under the spectral performance curve (AUSPC) serves as a metric of model generalizability and enables comparisons across models.

To generate a split with SPECTRA, a similarity definition and a SPECTRA parameter (SP) value between 0 and 1 are required. SP controls the level of cross-split overlap: values closer to 0 create splits resembling classical random splits, while values closer to 1 produce stricter splits with minimal or no overlap between train and test sets. For example, at an input of 1, no similar samples are shared between the train and test sets.

For eICU and MIMIC-IV, we define two patients as similar if: (1) they are of the same gender, (2) they are born in the same decade, and (3) they share at least one ICD-9 or ICD-10 category. We exclude ICD-9 and ICD-10 codes that are present in more than 50% of patients to avoid overly generic features. SPECTRA systematically prunes similar patients to produce splits. For this study, we generate 20 splits with SP values that are evenly spaced between 0 and 1. Given a train and test set, cross-split overlap is defined as the proportion of samples in the train set that are similar to at least one sample in the test set.

#### A.2.3. Constructing cohorts of patients with type 1 diabetes mellitus

To define a type 1 diabetes mellitus (T1D) patient cohort in eICU and MIMIC-IV, we identify patients with T1D and matched healthy individuals. A patient has T1D if the ICD-10 code E10 (or the equivalent ICD-9 code 250) is present in the electronic health records. Matched healthy patients are defined by three criteria. First, the patient must not contain any of the following ICD-10 (and ICD-9 equivalent) codes: E11, E13, E12, E08, E09, R73, and O24. An initial healthy patient cohort is constructed using these filtering codes. Next, we identify frequently co-occurring ICD codes between the initial set of patients and patients with T1D to filter out generic ICD codes (threshold = 20). Finally, healthy patients are matched with a T1D patient if: they are of the same gender, they are born in the same decade, and they share at least 50% of ICD codes.

## B. Further Implementation Details

We provide code and instructions in our GitHub repository to implement Clef, baselines, and ablations: https://github.com/mims-harvard/CLEF. For the implementation of baselines, we followed the authors’ recommendations on model design and hyperparameter selection from the original publications.

### B.1. Hyperparameter Sweep

For all models trained from scratch, the selection of hyperparameters are: dropout rate ∈ [0.3,0.4,0.5,0.6], learning rate ∈ [0.001,0.0001,0.00001], and number of layers (or blocks in xLSTM) ∈ [4,8]. As the number of heads must be divisible by the number of features, the number of heads for eICU (18 lab tests) ∈ [2,3,6,9] and for others ∈ [4,8]. For xLSTM, additional hyperparameters are: 1D-convolution kernel size ∈ [4,5,6] and QVK projection layer block size ∈ [4,8].

### B.2. Best Hyperparameters

#### MIMIC-IV dataset.

The best hyperparameters for models trained on the MIMIC-IV dataset are: dropout rate = 0.6, learning rate = 0.0001, number of layers (or blocks in xLSTM) = 8, and number of heads = 4. For xLSTM models, 1D-convolution kernel size = 4 and QVK projection layer block size = 4. For Clef models, the number of FNN in the concept encoder = 1 (Appendix Figures 8–9).

#### eICU dataset.

The best hyperparameters for models trained on the eICU dataset are: dropout rate = 0.6, learning rate = 0.0001, number of layers (or blocks in xLSTM) = 8, and number of heads = 6. For xLSTM models, the number of heads = 2, 1D-convolution kernel size = 4 and QVK projection layer block size = 4. For Clef models, the number of FNN in the concept encoder = 1 (Appendix Figures 8–9).

#### Waddington-OT (WOT) dataset.

The best hyperparameters for models trained on the WOT dataset are: dropout rate = 0.6, learning rate = 0.00001, number of layers (or blocks in xLSTM) = 4, number of heads = 8. For xLSTM models, 1D-convolution kernel size = 4 and QVK projection layer block size = 8. For Clef models, the number of FNN in the concept encoder = 0 (Appendix Figures 8–9).

## C. Additional Figures and Tables

**Table 2.** Generalizability of Clef, baselines, and ablations on eICU and MIMIC-IV datasets in immediate and delayed sequencing. Performance is measured by the area under the spectral performance curve (AUSPC) for MAE (Appendix Figure 11) or RMSE (Appendix Figure 12). Smaller AUSPC values indicate better performance.

Model	eICU				MIMIC-IV			
	Immediate		Delay		Immediate		Delay	
	MAE	RMSE	MAE	RMSE	MAE	RMSE	MAE	RMSE
Transformer	27.06 ± 0.98	59.83 ± 1.14	22.59 ± 1.21	50.29 ± 0.56	40.87 ± 0.15	71.77 ± 0.21	44.61 ± 0.19	80.38 ± 0.32
Clef-Transformer (FFN = 0)	15.16 ± 1.09	32.95 ± 2.47	14.36 ± 1.07	34.27 ± 2.12	32.79 ± 1.41	57.76 ± 3.39	35.65 ± 1.73	65.10 ± 4.43
Clef-Transformer (FFN = 1)	10.99 ± 0.31	27.57 ± 0.27	9.25 ± 0.60	27.69 ± 0.22	21.35 ± 3.16	36.92 ± 5.46	23.83 ± 3.26	44.11 ± 5.83

xLSTM	28.47 ± 0.63	62.28 ± 1.38	23.11 ± 0.91	52.53 ± 1.98	40.75 ± 0.30	71.90 ± 0.40	44.31 ± 0.24	80.38 ± 0.33
Clef-xLSTM (FFN = 0)	16.73 ± 2.16	35.43 ± 6.01	15.32 ± 2.10	34.68 ± 7.09	32.06 ± 1.13	53.42 ± 2.18	33.88 ± 1.98	57.73 ± 3.63
Clef-xLSTM (FFN = 1)	11.35 ± 0.11	28.09 ± 0.08	9.04 ± 0.18	26.21 ± 0.48	21.04 ± 2.32	37.50 ± 4.60	22.63 ± 2.61	42.12 ± 5.03

MOMENT	53.49 ± 0.03	90.54 ± 0.03	48.83 ± 0.02	82.50 ± 0.02	46.55 ± 0.01	77.22 ± 0.01	50.59 ± 0.02	85.72 ± 0.01
Clef-MOMENT (FFN = 0)	47.69 ± 0.33	82.18 ± 0.34	40.10 ± 0.44	72.70 ± 0.46	44.01 ± 0.35	73.83 ± 0.63	46.88 ± 0.38	81.20 ± 1.27
Clef-MOMENT (FFN = 1)	47.56 ± 1.60	82.81 ± 2.88	39.91 ± 1.65	72.54 ± 3.20	42.92 ± 0.52	70.72 ± 1.96	45.75 ± 0.65	77.35 ± 2.77
